# Women’s experience with peer counselling and social support during a lifestyle intervention among women with a previous gestational diabetes pregnancy

**DOI:** 10.1080/21642850.2019.1612750

**Published:** 2019-05-09

**Authors:** Meghan S. Ingstrup, Lisa A. Wozniak, Nonsi Mathe, Sonia Butalia, Margie H. Davenport, Jeffrey A. Johnson, Steven T. Johnson

**Affiliations:** a School of Public Health, University of Alberta, Edmonton, Alberta, Canada; b Departments of Medicine and Community Health Sciences, Cumming School of Medicine, University of Calgary, Calgary, Alberta, Canada; c Libin Cardiovascular Institute of Alberta, University of Calgary, Calgary, Alberta, Canada; d O’Brien Institute for Public Health, University of Calgary, Calgary, Alberta, Canada; e Program for Pregnancy and Postpartum Health, Physical Activity and Diabetes Laboratory, Faculty of Kinesiology, Sport, and Recreation, University of Alberta, Edmonton, Alberta, Canada; f Women and Children’s Health and Research Institute, Alberta Diabetes Institute, University of Alberta, Edmonton, Alberta, Canada; g Faculty of Health Disciplines, Athabasca University, Athabasca, Alberta, Canada

**Keywords:** Social support, gestational diabetes mellitus, physical activity, peer counselling

## Abstract

**Purpose:**

Women who are diagnosed with gestational diabetes mellitus have an increased risk of developing type 2 diabetes, but most receive little guidance regarding disease prevention. This study examined the role and usefulness of social support, including peer counselling in facilitating behaviour change as a part of a healthy eating and physical activity intervention among women with a previous gestational diabetes mellitus pregnancy.

**Methods:**

We used a qualitative descriptive approach to investigate participants’ experiences with the social support they received during the intervention. We used purposeful sampling and invited women and peer counsellors to participate in semi-structured interviews. Data were analyzed using content analysis.

**Results:**

We interviewed nine women and two peer counsellors. Participants received emotional, appraisal, and informational types of social support from the peer counsellor and exercise specialist that they reported as useful. Additionally, participants’ received informal emotional and instrumental support from family, friends, and colleagues that they found useful in supporting behaviour change.

**Conclusions:**

Different types of social support are important to encourage behaviour change. These findings provide insight into the types of social support women with previous gestational diabetes mellitus find useful, in addition to practical ways that social support can be incorporated into future interventions.

## Introduction

Gestational diabetes mellitus (GDM) is a common complication of pregnancy that increases a woman’s risk for developing type 2 diabetes later in life (Buchanan, Xiang, Kjos, & Watanabe, 2007; Ferrara, Hedderson, Quesenberry, & Selby, 2002). While individual studies vary, the meta-evidence demonstrates there are benefits associated with being physical active and eating healthfully, with greater benefits the earlier these lifestyle changes are adopted (Bain et al., 2015; Jones, Roche, & Appel, 2009; Russo, Nobles, Ertel, Chasan-Taber, & Whitcomb, 2015; Sanabria-Martinez et al., 2015). The heterogeneity in the literature is possibly due to behaviour-change barriers, such as lack of knowledge about recommended guidelines for physical activity and healthy eating and/or a lack of social support for engaging in these healthful behaviours (Eades, France, & Evans, 2018; Jones et al., 2009; Rees et al., 2017).

There is an absolute need to provide women with previous GDM access to information and support regarding type 2 diabetes prevention (Gabbe, Landon, Warren-Boulton, & Fradkin, 2012). Postpartum women are faced with unique challenges due to time constraints and barriers such as childcare needs; however, access to social support during lifestyle changes may mitigate some of those barriers (Nicklas et al., 2011; Razee et al., 2010; Smith, Wah Cheung, Bauman, Zehle, & McLean, 2005). Emerging research indicates that the provision of social support may be beneficial for women engaging in behaviour change in comparison to those with little or no access to social support (Kim, McEwen, Kieffer, Herman, & Piette, 2008; Smith et al., 2005). However, research investigating the type(s) and implementation of social support for effective behaviour change post GDM is in its infancy. Therefore, we included social support through peer counsellors and an exercise specialist during a 24-week lifestyle intervention, called Healthy Eating and Active Living after Gestational Diabetes Mellitus (HEALD-GDM) (Johnson et al., 2017), to address some of the barriers women face when engaging in healthy eating and active living after a recent GDM pregnancy. Peer counsellors were trained in behaviour change theory (i.e. Social Cognitive Theory), and the topics they discussed with participants were designed to address core constructs of Social Cognitive Theory including knowledge, perceived self-efficacy, outcome expectations, goals, perceived facilitators, and potential barriers (Bandura & Walters, 1977; Johnson et al., 2017).

The literature on social support is vast, with a variety of different definitions. For the purposes of this study, we focused on the four most prominent types of social support: (a) emotional; (b) appraisal; (c) informational; and, (d) instrumental (House, 1981). Emotional support is conceptualized as expressions of caring, concern, empathy, and listening, while appraisal support offers evaluations and positive affirmations regarding ability and internal value, and can result in feelings of enhanced worth (Malecki & Demaray, 2003). Informational support includes opinionated and factual advice and guidance, whereas instrumental support is viewed as the provision of material and tangible aid and services (Cutrona & Suhr, 1992; House, 1981). Because the 24-week lifestyle intervention operationalized different forms of social support through peer counsellors and an exercise specialist (Johnson et al., 2017), we aimed to understand participants’ perspectives regarding the role and usefulness of social support, in promoting behaviour change (e.g. increasing physical activity and healthy eating) six-months after the intervention was completed.

## Methods

The details of the intervention are described in detail elsewhere (Johnson et al., 2017). Briefly, the intervention randomized women (with previous and recent GDM pregnancy, aged 18–45, capable of sustained walking, and able to understand English) into a 24-week lifestyle modification programme led by an exercise specialist or a control condition (i.e. received generic public health guidelines for healthy eating and active living). Participants in the lifestyle programme received an initial 4 h of instruction led by a certified Exercise Specialists at local recreational facilities, followed by theory-guided peer-led telephone support. Our primary outcome was objectively derived weekly moderate and vigorous physical activity. Participants who completed the lifestyle modification programme and peer counsellors were invited to participate in a semi-structured interview regarding experiences of social support throughout the intervention.

We used qualitative description (Sandelowski, 2000, 2010) to elicit participants’ and peer counsellors’ perspectives on the role and usefulness of social support in promoting physical activity (i.e. walking) and healthy eating. Qualitative description is an appropriate approach when describing and summarizing a phenomenon (Neergaard, Olesen, Andersen, & Sondergaard, 2009; Sandelowski, 2000), such as the role and usefulness of social support in this study. Using a qualitative approach to investigate lifestyle interventions provides complementary data to quantitative research that will further inform future interventions.

### Data collection

We purposefully sampled participants from the HEALD-GDM intervention arm who had participated in at least one of four group sessions with the exercise specialist and five or 25% of peer counselling sessions (i.e. of the 20 possible). Of the 13 women who met our criteria and were contacted, 9 agreed to participate (69% response rate). Due to the documented challenges of recruiting in this population (Smith et al., 2005) we also invited the peer counsellors to participate in interviews as additional key informants, with 3 out of 4 peer counsellors participating (75% response rate). A key informant is an individual who can provide information on a particular experience (Marshall, 1996). It is important to note that our sampling technique is not meant to be representative of a population; rather it is representative of individuals who are experts regarding a similar experience, such as social support after GDM (Marshall, 1996; Morse, 1991; Rosal, Lemon, Nguyen, Driscoll, & DiTaranto, 2011; Yardley, 2007).

We invited participants and peer counsellors via email to participate in a telephone interview. A trained researcher in qualitative methodologies (MI) conducted the interviews using semi-structured interview guides, which were refined as data analysis progressed. Types of guiding interview questions included: Tell me about your experience participating in the programme, including your interactions with the peer counsellor and/or other women in the programme/Tell me about your experience as a peer counsellor; Who can you count on to support you to walk more and eat healthily?; and Describe examples of support you received/or gave. Interviews ranged from 15 to 30 min, were digitally recorded and transcribed for subsequent analysis, and verified for accuracy by MI. In addition, MI completed notes immediately after each interview to record emerging ideas and personal reflections (Yardley, 2007). Audio files were transcribed verbatim and verified for accuracy. We assigned pseudonyms to all participants to protect their identity.

### Data analysis

We used content analysis (Forman & Damschroder, 2007) to systematically code and organize the data, as is appropriate for qualitative descriptive studies (Sandelowski, 2000). One researcher (MI) conducted the primary analysis using an inductive approach (Sandelowski, 1995). First, audio recordings were reviewed and transcript data were read and re-read to get a sense of the data as a whole; memos were kept to record early observations and refined throughout the analytic process. Next, we used an inductive approach to develop codes that emerged from the data itself related to the study aim. Using an iterative process, codes and code definitions were refined and organized into emerging themes. Regular research team meetings were held to review codes, code definitions, and emerging themes and to discuss discrepancies to reach consensus. As is standard in qualitative inquiry, we conducted concurrent data collection and analysis until data saturation was achieved; that is, the major concepts were well defined and explained and no new concepts or themes were expected to emerge from further examination (Morse, 1995). Data were managed using ATLAS.ti Version 7 software( 2012).

### Ethics Statement

The University of Alberta Health Ethics Research Board approved this study and all participants provided informed consent (HREB reference #: Pro00051650).

## Results

We conducted telephone interviews with 11 female respondents, including nine participants and two peer counsellors, between January and August 2017. All of the participants identified as Caucasian/European, were married, and had experienced a GDM pregnancy within the last 18 months. The average age of the participants was 38 years, the majority (78%) had undergraduate university degrees and approximately half (55%) had two or more children in their household. We did not collect demographic information on the peer counsellors as they were not involved as participants in the trial, however it should be noted that all the peer counsellors had a history of GDM.

Participants identified several types of support throughout two sources, including: emotional, appraisal, and informational sources, through HEALD-GDM (e.g. peer counsellors, exercise specialist, and other intervention participants) and emotional and instrumental support from their broader social network (e.g. family, friends, and colleagues). However, participants reported variable usefulness of support offered by these sources, with some explaining that they did not receive the social support they felt they needed to facilitate behaviour change. Finally, participants provided feedback regarding peer counsellor qualities that they thought would be effective in the provision of social support, including being knowledgeable and engaging. We detail the findings below with illustrative quotes. For all quotes, we include the respondent group (e.g. participant pseudonym or peer counsellor), and age of participant; however, we do not provide the age of peer counsellors as we did not collect their demographic information.

### Variable usefulness of available social support

Many participants agreed that some type of formal social support, such as that provided from the HEALD-GDM intervention was important to promote behaviour change. Intervention-related support was *most useful* to participants when it was offered weekly to facilitate accountability, offer motivation (i.e. appraisal support), encouragement (i.e. emotional support), and opportunities for guidance (i.e. informational support).

Many participants explained that it was useful when peer counsellors held them accountable to the intervention and their individual goals through consistent, weekly support via telephone calls. Kylie (age 34) reflected on how peer counselling ‘made me more accountable. It drove me to try and reach my goals’ and how ‘it was nice that there was that availability of somebody to be accountable to every week’. The emotional and appraisal support provided by the peer counsellors allowed participants to discuss their weekly progress and provided motivation to stay engaged in physical activity and their behaviour change goals.

In addition, over half of participants explained that the appraisal and emotional support offered by the exercise specialist was motivating or encouraging and thus promoted their involvement in HEALD-GDM, particularly at the beginning of the intervention. For example, Debby (age 45) explained:

The encouragement of [the exercise specialist], who did my assessments, she has a level of enthusiasm that makes me believe you can do this, and she was quite a key ingredient to the success of the program and for my engagement in it.

Interestingly, the exercise specialist’s main role in the intervention was to provide informational support, not emotional or appraisal support to participants. Regardless, it appears that her ability to encourage and engage with participants influenced their motivation and attitude, which may have influenced participants’ perceived success in achieving their goals.

Several participants said that it was useful when peer counsellors provided informational support to help them identify potential and existing barriers to behaviour change, find personalized solutions, and set realistic expectations*.* For example, some participants found it helpful when peer counsellors provided strategies for increasing their daily steps when they were having difficulty finding time to engage in regular physical activity. Kassi (age 43) explained: ‘Generally, if I had set goals for the week, [the peer counsellor asked] “how it’s going? What could come against those goals? [And] what I could do to mitigate whatever might be putting my goals at risk?”’ Additionally, a peer counsellor explained how she helped participants set realistic goals: ‘You kind of have to re-adjust and re-evaluate what is possible for them because some people set unrealistic expectations going into the program’ (Peer counsellor 1). Therefore, the provision of informational support was useful in setting, and maintaining goals by identifying barriers and possible solutions.

It was anticipated that a minimal level of group cohesion would form between the participants during the four group-based sessions with the exercise specialist over the course of the intervention; however, it appears that participants desired emotional and instrumental support from their peers that was different from support offered from peer counsellors. For example, some participants discussed attempts to facilitate supportive relationships with other participants outside of the intervention proper; however, this proved difficult to achieve. In an attempt to build a social network, participants communicated with each other via text message and Facebook to provide words of encouragement and support and to establish a walking/support group; however, neither regular communication nor the establishment of a walking group appeared to last over the duration of the intervention. For example, some participants could not participate in a walking support group due to geographical barriers.

So [another intervention participant] and I text each other back and forth, [to ask] “how are you doing? Are you getting your steps in?” But … because we’re so far apart we never really were like “hey, let’s meet up”, but if we did – if I was [geographically] closer with a group of moms it would be much easier for sure. (Shannon, age 33)

Two participants were successful in providing substantial support to each other throughout the intervention. They attended the intervention appointments and engaged in physical activity together (i.e. instrumental support), and relied on each other for emotional support (e.g. listened, offered words of encouragement), which they reported as helpful in achieving their goals.

While some respondents reported positive experiences with the support provided by the intervention, others described *less useful support* due to inconsistent interactions or a lack of rapport with the peer counsellor, both of which likely led to a perceived lack of social and emotional support. Regarding inconsistent interactions, Laura (age 37) described intermittent peer counsellor contact throughout the intervention: ‘It didn’t really start in the beginning and didn’t end at the end of the research. It was just a chunk in the middle and then that piece was done’. Lack of rapport was another experience participants listed as less useful for social support. For example, Kylie (age 34) explained: ‘We didn’t click on that level where we [peer counsellor] would expand our conversation outside of what was required’. Indeed, when rapport was not established, it negatively influenced the quality and depth of conversation between peer counsellors and participants, as explained by Mariah (age 36):

Well that was the thing, we really didn’t discuss it, so for me to relate with the person I need to know what their situation was, like she mentioned she had [GDM] and that’s why she was part of the peer counselling, but really we didn’t connect on that level that I felt comfortable talking to her about my situation, so I would feel comfortable if it [was] someone I could relate to and we could have a really good conversation about it.

In summary, there were discordant opinions regarding the degree of, and usefulness of emotional, appraisal, and informational support available during the intervention to assist with behaviour change. Initially, the exercise specialist provided emotional and appraisal support through encouragement and motivation. Thereafter, participants found intervention-related support most useful when the exercise specialist provided emotional and appraisal support through motivation and encouragement and when peer counsellors provided emotional and appraisal support through encouragement and motivation and informational support through barrier identification on a weekly basis. Beyond intervention-specific support, a number of participants provided emotional and instrumental support for each other intermittently during the intervention; however, most participants experienced geographical barriers that prevented meaningful engagement in physical activity with each other outside of the four intervention group sessions.

### Availability of and usefulness of social support from social networks

Many participants identified members of their larger social network such as family, friends, and colleagues as sources of social support that complimented support provided through the intervention. In addition, some participants reported that they sought out support from their social network when their peer counsellor was unavailable to provide the social support that they required. This complementary support they received from their social networks was important for promoting behaviour change. Specifically, participants reported that their social network support was most useful when they provided encouragement (i.e. emotional support) and motivation (i.e. appraisal support), and helped with childcare and engaged in physical activity with them (i.e. instrumental support).

For many participants, partners provided substantial support that facilitated intervention engagement. For example, Debby (age 45) said: ‘My husband was my main level of support and encouragement to get involved in the program’. Audrey (age 32) summarized the importance of regular support received from her larger social network: ‘My spouse, my extended family, and my group of friends. Everyone knew I was [going to] take part in the study and so they check in and ask about how that’s going, and that made a difference for me’.

Some participants described receiving instrumental support in the form of childcare, which opened opportunities for them to engage in physical activity. For example, Mariah (age 36) explained: ‘My husband he’s very active as well, so he’s always encouraging me to work out, gives me the time to do that, he picks my son up and then I work out’. By engaging in physical activity himself, perhaps this partner had insight into the necessity of childcare which prompted him to provide his partner with the time to achieve her activity goals.

Over half of participants explained that friends and colleagues provided instrumental support by engaging in physical activity with them, which helped participants work towards their physical activity goals. Kassi (age 43) said: ‘I have several friends, one of which is a colleague that I’ve recently started jogging with’. Another participant said: ‘my social network really helped, and [they were excited] and there were people to walk with me’ (Audrey, age 32). Hence, some participants benefited from having members of their social network engage in physical activity with them.

A few participants indicated that their children were sources of motivation, and in some cases, provided instrumental support for behaviour change because they wanted to teach their children healthy lifestyle habits. Selena (age 44) explained this when she said: ‘Because I have a 3 year-old son and an 8 year-old daughter, I’m always making healthy choices and eating with them’. Participants were aware that, as parents, they were role models for healthy behaviours, which was an additional source of motivation as they strove to teach their children healthy lifestyle habits.

Unfortunately, not all participants reported receiving useful support for behaviour change from their larger social network. Specifically, social network support was less useful when members were dismissive of their involvement in the intervention or were unable to provide support due to geographical barriers. For example, despite discussing their involvement in the intervention, some participants reported that their broader social network was too busy to offer support or were dismissive of their decision to engage in behaviour change initiatives:

My friends and family, I could tell them that I’m doing it [the intervention] but they’re busy with their own stuff and they wouldn’t go out and walk with me. Or my mom [is] diabetic. She’s like, “Ah, you did enough walking. You don’t need to do any more”. I’m like, “Mom, you know, that’s the whole point of this is that I need to get more so I don’t have to take medicine like you do”. (Taylor, age 36)

Selena (age 44) described a similar experience with her partner: “My husband is great, but when it comes to fitness and healthy stuff and everything else he’s one of those that would say, “Sit down and have a cheeseburger with me”, right? So I needed [my peer counsellor]’. It is possible that some people do not understand the commitment required to successfully accomplish behaviour change, or why women who have experienced a GDM pregnancy would want to increase their physical activity and healthy eating resulting in a dismissive behaviour; however, participants did not find this type of behaviour helpful as they attempted to adopt a more physically active lifestyle.

Additionally, a number of participants explained that friends and family were unable to engage in physical activity with them because they lived far away:

I don’t have a lot of friends who live near me who I could walk with. I don’t have family that lives in Canada. I have friends here, but we don’t live in the same sort of neighbourhood. They were vaguely encouraging but I’m not in the same sort of place. (Debby, age 45)

As geographical barriers are tough to overcome, it was difficult for individuals who live far away to provide instrumental support to participants.

### ‘Peer’ counsellor qualities and social support

To further our understanding of the role of social support, we asked participants to describe the qualities of peer counsellors necessary to provide useful social support. Participants explained that it was more important that peer counsellors were knowledgeable about GDM and the barriers to behaviour change, were empathetic about the realities of being a parent, and engaged, rather than sharing specific ‘peer’ experiences, including being a parent, having had GDM, or being female. For example, Taylor (age 36) said she did not believe that the individual providing support had to have had GDM: ‘I don’t think it’s necessary [to have had GDM] but I do think that some kind of knowledge about it – so whether they studied it or went through it’. Additionally, Mariah, (age 36) said:

I think it’s the motivation and [ability to] be an engaging person to coach. I don’t think they had to have gestational diabetes, I think it’s someone who wants to help others be healthy and active and lead a good life.

In relation to parental experience, Debby (age 45) explained, ‘It would be possible [to be a peer counsellor and not a parent] but I think they’d need to have a fairly good understanding of the dynamics of being a primary caretaker of children’. In regard to the gender of the peer counsellor, Laura (age 37) explained: ‘For me that wouldn’t matter. It’s the same thing, right? I think it becomes about the knowledge. As long as they know what they’re talking about and can kind of offer that support I think it would be fine’. Thus, it was important to participants that peer counsellors have the ability and knowledge to effectively engage with, empathize, and provide support during behaviour change initiatives.

## Discussion

This study examined perceptions regarding the role and usefulness of emotional, appraisal, informational, and instrumental social support to promote behaviour change in women with previous GDM. Given that lack of social support is routinely reported as a barrier to behaviour change among women in the postpartum period, the findings of this study contribute to the literature by not only identifying who, but also what types of social support are relevant to support healthy eating and active living. We suggest interventions should consider who can offer support but also what types of support when considering lifestyle changes to reduce the risk of developing type 2 diabetes. Overall, our findings indicated that most participants received some level of social support that was useful for supporting behaviour change, despite some dissatisfaction regarding the lack of emotional and informational support through peer counselling and/or instrumental and emotional support from their own social network.

In addition, participants’ accounts indicate that the peer counsellors operationalized the constructs from Social Cognitive Theory (SCT) that were targeted through the HEALD-GDM intervention (Johnson et al., 2017). Components of the SCT framework were most influential for participants when peer counsellors provided regular (i.e. weekly) support, provided information, identified barriers and possible solutions, and offered positive affirmations that encouraged and motivated participants towards goal achievement (i.e. emotional, appraisal, and informational support). Thus, our findings provide support for the use of SCT as a framework to guide the provision of social support for behaviour change in this population (Bandura, 2004; Ferrara et al., 2011). However, we suggest a more vigorous selection and training process for individuals whose role is to provide social support to increase the likelihood of sufficient support for intervention participants (Philis-Tsimikas et al., 2014).

Rapport emerged as an important factor that influenced the success of the participant-peer counsellor relationship and, therefore, the usefulness of social support provided. When rapport was established by the peer counsellors, the peer counselling component was a useful source of social support. Conversely, if rapport was not established, participants reported limited emotional, appraisal, and informational support and/or inconsistent or limited interactions with the peer counsellor. The importance of rapport has been reported in previous research where established rapport was associated with improved treatment outcomes, whereas a lack of rapport resulted in poor outcomes for a variety of psychological phenomenon (e.g. depression, alcoholism, & post-traumatic stress disorder) (Joe, Simpson, Dansereau, & Rowan-Szal, 2001; Leach, 2005). As such, we emphasize the importance of equipping those individuals providing support with the skills to establish and maintain rapport for future interventions with this population.

Several participants received informal, emotional, and instrumental support outside of that offered through the intervention from family, friends, and colleagues, including encouragement and company for engagement in physical activity. Interestingly, some participants discussed that they wanted to set a good example for their children, and therefore they engaged in healthy eating and physical activity with their children. This demonstrates a seemingly mutually beneficial relationship through which the women received motivation to eat healthy and be more physically active, while modelling and engaging children in these behaviours. Motivation for behaviour change due to concern for their child’s health was also reflected in a recent study that found women made dietary and physical activity changes to protect the health of their unborn child (Eades et al., 2018). Thus, there may be some merit in finding ways to include children in behaviour change initiatives, which might alleviate the barrier of childcare, and promote a healthier lifestyle for both parties (Rosal et al., 2011).

Other participants reported inadequate emotional and instrumental support from their social network including dismissive comments or geographical barriers. These findings highlight an issue among this population regarding social support for chronic disease management where family and friends were insensitive towards, or unable to help alleviate challenges with disease prevention and management (Clark & Nothwehr, 1997). Thus, those looking to implement future interventions should consider how to equip the family and friends of participants with information regarding the provision of useful support for those engaging in preventative or management behaviours towards chronic diseases.

The risk of a type 2 diabetes diagnosis among women with previous GDM can be decreased with dietary and physical activity modifications (Johnson et al., 2016). However, there are unique challenges associated with this population, such as lack of time or childcare needs that create barriers to participation in physical activity and healthy eating (Smith et al., 2005), which was also reflected in our findings. Geographical location was discussed as a barrier for the provision of instrumental social support from other intervention participants and members of participants’ social network. We tried to moderate the issue of childcare by offering free childcare during appointments with the exercise specialist; however, some participants needed childcare on a regular basis in order to participate in walking on their own. Peer counsellors were able to help participants find ways to mitigate some of the time barriers by providing time management advice and realistic goal setting; regardless some participants still had difficulty finding time. Lack of time and access to childcare remain significant barriers for this population and future studies should explore ways to address these barriers to meet the needs of this population.

Our study has several strengths including providing in-depth insight into women’s experiences of social support to promote behaviour change after GDM, which has furthered our understanding and allowed us to provide informed, practical suggestions for future interventions. However, our work does have limitations. Our sample size may be considered small even based on standards in qualitative research. Perhaps this is not surprising given that women with GDM are notoriously difficult to recruit for research, and they face unique barriers such as lack of time and childcare needs that limit and restrict participation (Smith et al., 2005). It is important to note that the voices and experiences of this population are desperately needed to contribute to the literature, which our study accomplished. In addition, we did not include the members of the women’s social network because we were interested in the perspectives of women with previous GDM and their peer counsellors; however, the perspectives of partners, friends, and family could have yielded additional information about the role and usefulness of social support for this population. Lastly, as with all qualitative research, there is limited transferability of our results beyond the current context (i.e. sample and setting). However, it is reasonable to assume that our findings are applicable to the development of future interventions for this population.

In conclusion, the findings of this study contribute to the literature by informing practical ways through which interventions can provide different types of social support to this population. Regardless of the respondents’ experiences with the social support provided by the HEALD-GDM intervention, most participants agreed that some kind of formal intervention-provided support including accountability, motivation, and counselling was important to promoting behaviour change. The telephone peer counselling component was useful in the provision of emotional, appraisal, and informational support when rapport was established and the use of SCT framework helped peer counsellors guide participants through goal setting and identify barriers and provide possible solutions. However, some participants required additional in-person support (instrumental support) and sought out individuals who could engage in physical activity with them outside of the intervention. Therefore, questions such as who is best suited to be a peer counsellor or to provide social support, how best to train them, and how much support is the right amount remain as future research directions.

## Availability of data and material

The datasets generated and/or analyzed during the current study are not publicly available due to confidentiality but are available from the corresponding author on reasonable request.
